# Supplementary material for: Active site specificity profiling datasets of matrix metalloproteinases (MMPs) 1, 2, 3, 7, 8, 9, 12, 13 and 14
Source: Data Brief. 2016 Feb 22;7:299–310. doi: 10.1016/j.dib.2016.02.036 (PMC4777984; doi:10.1016/j.dib.2016.02.036)
Supplement: Supplementary file 10 — Supplementary material [file mmc10.zip › WebPICS_hMMP2_G_1%/subsite_coop.html]

 

PICS results


- # About
- # Manual
- # Analysis
- # Results

- Redraw
- Seqlogo
- Dependency
- Coop

**Potential subsite cooperativity analysis for hMMP2\_G\_1%**   
Minimum difference for subsite dependency was set to +- 10 percentage-points  
Subsite dependency was checked for all positional occurences above 1 x natural abundance  
**Subsite dependency check is restricted to P3 - P3'**

|  |  |  |  |
| --- | --- | --- | --- |
| Fixed residue | Affected residue(s) | Change (percentage-points) | Vice-Versa change |
| P3\_A | P1\_A | 13.5 | 13.1 |
| P3\_I | P1\_G | 12.3 | 10.9 |
| P3\_I | P1\_N | 11.9 | 17.0 |
| P3\_I | P2prime\_N | 20.1 | 66.6 |
| P3\_I | P3prime\_V | 19.3 | 23.6 |
| P3\_L | P1\_N | 13.8 | 15.5 |
| P3\_L | P2prime\_Y | 12.1 | 39.4 |
| P3\_L | P3prime\_S | 19.2 | 13.1 |
| P3\_P | P1prime\_V | 16.8 | 50.2 |
| P3\_V | P2prime\_K | 15.0 | 14.0 |
| P3\_V | P3prime\_K | 20.3 | 32.6 |
| P2\_Q | P2prime\_I | 24.7 | 12.7 |
| P2\_Q | P2prime\_Q | 11.0 | 14.1 |
| P1\_D | P3prime\_N | 10.9 | 13.3 |
| P1\_G | P2prime\_N | 17.5 | 65.0 |
| P1\_G | P3prime\_V | 18.7 | 25.7 |
| P1\_H | P3prime\_K | 35.6 | 13.9 |
| P1\_N | P2prime\_F | 11.3 | 17.4 |
| P1\_N | P2prime\_Q | 11.7 | 19.3 |
| P1\_Q | P2prime\_M | 15.4 | 24.1 |
| P1\_S | P2prime\_I | 19.1 | 16.4 |
| P1prime\_C | P2prime\_T | 29.4 | 11.7 |
| P1prime\_I | P3prime\_G | 16.2 | 14.0 |
| P1prime\_M | P2prime\_M | 17.2 | 24.5 |
| P1prime\_W | P2prime\_Y | 16.7 | 10.5 |
| P2prime\_I | P3prime\_N | 15.6 | 30.2 |
| P2prime\_K | P3prime\_K | 12.1 | 20.7 |
| P2prime\_N | P3prime\_V | 79.0 | 29.2 |

  
**PICS analysis of protease: hMMP2\_G\_1%**  
246 cleavage sites analyzed  
PICS library made with (T)rypsin, (G)luC or (C)hymotrypsin:   
Cutoff for graphic display: 2 x natural abundance  

|  |  |
| --- | --- |
| Positional occurences  (table for total and relative (in %) values) | Occurences relative to natural abundance (table) |
|  |  |
